# Optogenetic-enabled biomanufacturing of long-projecting motor tracts for neural reconstruction

**DOI:** 10.1088/1758-5090/aea7dc

**Published:** 2026-10-13

**Authors:** Justin C Burrell, Vishal J Tien, Phuong T Vu, David R Clizbe, Michael Spadola, Yohannes Ghenbot, Ali K Ozturk, D Kacy Cullen

**Affiliations:** 1 Center for Brain Injury & Repair, Department of Neurosurgery, Perelman School of Medicine, University of Pennsylvania, Philadelphia, PA, United States of America; 2 Center for Neurotrauma, Neurodegeneration & Restoration, Corporal Michael Crescenz Veterans Affairs Medical Center, Philadelphia, PA, United States of America; 3 Center for Innovation & Precision Dentistry, Penn Dental Medicine, University of Pennsylvania, Philadelphia, PA, United States of America; 4 Department of Oral & Maxillofacial Surgery, Penn Dental Medicine, University of Pennsylvania, Philadelphia, PA, United States of America; 5 Department of Bioengineering, School of Engineering & Applied Science, University of Pennsylvania, Philadelphia, PA, United States of America

**Keywords:** neural tissue engineering, optogenetics, engineered micro-tissue, biomanufacturing, axon outgrowth

## Abstract

Tissue-engineered spinal tracts (TE-STs), a subclass of micro-tissue-engineered neural networks, are designed to replace long-range axonal pathways but are limited by the slow intrinsic growth of many neuronal subtypes, particularly spinal motor neurons. Optogenetic stimulation provides cell-type–specific, precise control of activity that may accelerate neurite extension during biofabrication. Accordingly, this study evaluates whether integrating optogenetic stimulation into the biomanufacturing process enhances motor axonal outgrowth within tailored hydrogel micro-columns *in vitro.* Motor neuron aggregates from embryonic rat spinal cord were generated by forced aggregation and encased within agarose micro-columns (701 *µ*m OD, ∼300 *µ*m ID; 3–12 mm length) to create TE-STs. Neurons were transduced to express the red-shifted channelrhodopsin ChrimsonR. Stimulation parameters were first optimized in planar cultures, where a 1-hour red-light train at 10 Hz (625 nm) maximized neurite outgrowth without significant heating. TE-STs were then stimulated at 10 Hz for 1 h at 7 d *in vitro* (DIV) and then fixed at 21 DIV. Groups included transduced and non-transduced neurons, each with and without light stimulation. Axonal extension was quantified within the micro-column lumen, and two-way analysis of variance was used for analysis. TE-STs exhibited robust axonal growth with organized tract architecture, preserved neuronal health, and stable motor neuron phenotype. Transduced neurons with and without light stimulation extended axons faster than non-transduced controls, whereas no differences were observed between non-transduced neurons with or without light. Notably, transduced neurons exposed to a single round of optogenetic stimulation at 7 DIV reached ∼5.5 mm of growth by 21 DIV, representing a ∼2.5-fold increase over baseline controls. Brief, targeted optogenetic activation is sufficient to accelerate axonal growth of engineered spinal motor tracts during *in vitro* biomanufacturing. These findings establish optogenetic stimulation as a programmable biomanufacturing input that accelerates axonal assembly through activity-dependent mechanisms while remaining compatible with scalable three-dimensional tissue fabrication.

## 1. Introduction

Repairing the nervous system after traumatic injury requires re-establishing long-distance axonal connections between disconnected neuronal populations. Achieving this is particularly challenging in the central nervous system (CNS), where intrinsic regenerative capacity is limited, and the post-injury microenvironment rapidly becomes inhibitory [1–5]. By contrast, the peripheral nervous system (PNS) retains a robust regenerative potential and can support axonal regrowth across centimeter-scale distances, providing a biological benchmark for the scale of reconnection required to restore meaningful function [6]. Translating these principles to the CNS, however, remains a major unmet challenge due to the combined effects of neuronal atrophy, glial scar formation, and growth-inhibitory extracellular matrix molecules such as chondroitin sulfate proteoglycans. Collectively, these degenerative and inhibitory sequelae, coupled with the absence of substantial spontaneous regeneration, result in the devastating and often permanent deficits observed following spinal cord injury (SCI).

To overcome these barriers, a wide range of strategies has been explored, including biomaterial scaffolds designed to guide regenerating axons, deliver trophic support, or promote remyelination [7–18]. Neuronal replacement approaches have also been investigated as a means to restore connectivity between injured and intact regions of the spinal cord [19–23]. Despite encouraging advances, robust regeneration of long descending tracts and durable functional recovery remain limited in preclinical SCI models, highlighting the need for strategies that address both structural and biological constraints on axonal growth.

A critical and often underappreciated bottleneck in neural repair and tissue engineering is the intrinsic limitation on axonal extension in conventional *in vitro* systems. In three-dimensional collagen-based cultures, spinal motor neuron neurite outgrowth typically remains confined to the sub-millimeter range over several days *in vitro*, even with optimized trophic support, with reported longest neurite lengths on the order of several hundred micrometers [24]. Similarly, while two-dimensional cultures can support longer neurite extension, reported axonal lengths commonly plateau at approximately 1–2 mm over weeks of culture [25, 26]. In more complex three-dimensional microfluidic or organotypic platforms, increases in axonal length are often reported as cumulative network length rather than single-axon span, reflecting increased axon number and branching rather than centimeter-scale elongation of individual axons [27]. Together, these findings underscore a persistent limitation of existing *in vitro* systems: while permissive matrices and trophic cues can enhance neurite growth, they rarely generate long, aligned axonal tracts suitable for bridging clinically relevant injury gaps.

Over the past decade, structured engineered neural tissues have emerged as a promising approach to address this architectural limitation. In particular, anatomically inspired micro-tissue engineered neural networks (*μ*TENNs) were developed to replace both lost neuronal populations and their long-projecting axonal connections within a single implantable construct [28–34]. *μ*TENNs consist of discrete neuronal populations connected by aligned axonal tracts encased within a protective hydrogel micro-column, enabling predefined geometry and directional connectivity. This strategy has been successfully applied to cortical-thalamic and nigrostriatal pathway reconstruction in rodent models [29, 32]. More recently, *μ*TENNs composed of dorsal root ganglia (DRG) sensory neurons have been deployed in rodent SCI models, where centimeter-scale preformed axonal tracts survived implantation, bridged lesion cavities, and extended new projections into host parenchyma. Notably, host axons were observed in proximity to implanted sensory axons, suggesting regeneration along graft-derived tracts through a mechanism analogous to axon-facilitated axonal regeneration (AFAR), previously characterized in the PNS [35, 36].

Despite these advances, the biomanufacturing of long neural constructs remains constrained by the slow intrinsic rate and limited extent of axonal growth exhibited by many neuronal subtypes, including spinal motor neurons (MNs) [25, 26, 37–39]. These growth limitations prolong fabrication timelines and restrict construct length, limiting scalability and translational applicability. Activity-dependent stimulation represents a promising strategy to overcome these constraints. Optogenetic activation has been shown to enhance neurite outgrowth of sensory neurons *in vitro* and to promote axonal regeneration and functional recovery following SCI *in vivo* [40, 41]. Beyond the brain, optogenetic approaches have been extended to spinal and peripheral circuits, providing a framework for integrating optical modulation into regenerative strategies. Recent studies demonstrate that direct optical stimulation of injured spinal circuits can promote new axonal growth and skilled forelimb recovery in sub-chronic injury models [42]. Moreover, combining optogenetic stimulation with neural progenitor grafts enhances graft integration and functional recovery in preclinical SCI models, underscoring the translational potential of optical modulation before and after implantation [43].

The present study builds upon this emerging body of work by introducing an optogenetically driven biomanufacturing paradigm for the generation of spinal motor neuron micro-tissues designed to reconstruct long spinal pathways (figure 1). Preformed constructs containing spinal MNs with aligned, long-projecting axonal tracts encapsulated within hydrogel micro-columns, termed tissue-engineered spinal tracts (TE-STs), are fabricated and characterized *in vitro*. The results demonstrate that optogenetic stimulation accelerates axonal extension during TE-ST fabrication, enabling the generation of long-projecting motor tracts within a shorter and more translationally relevant timeframe. Collectively, these findings establish optogenetic stimulation as a programmable input for neural tissue biomanufacturing and position TE-STs as a promising platform for restoring long-distance connectivity following neurotrauma.

**Figure 1.**
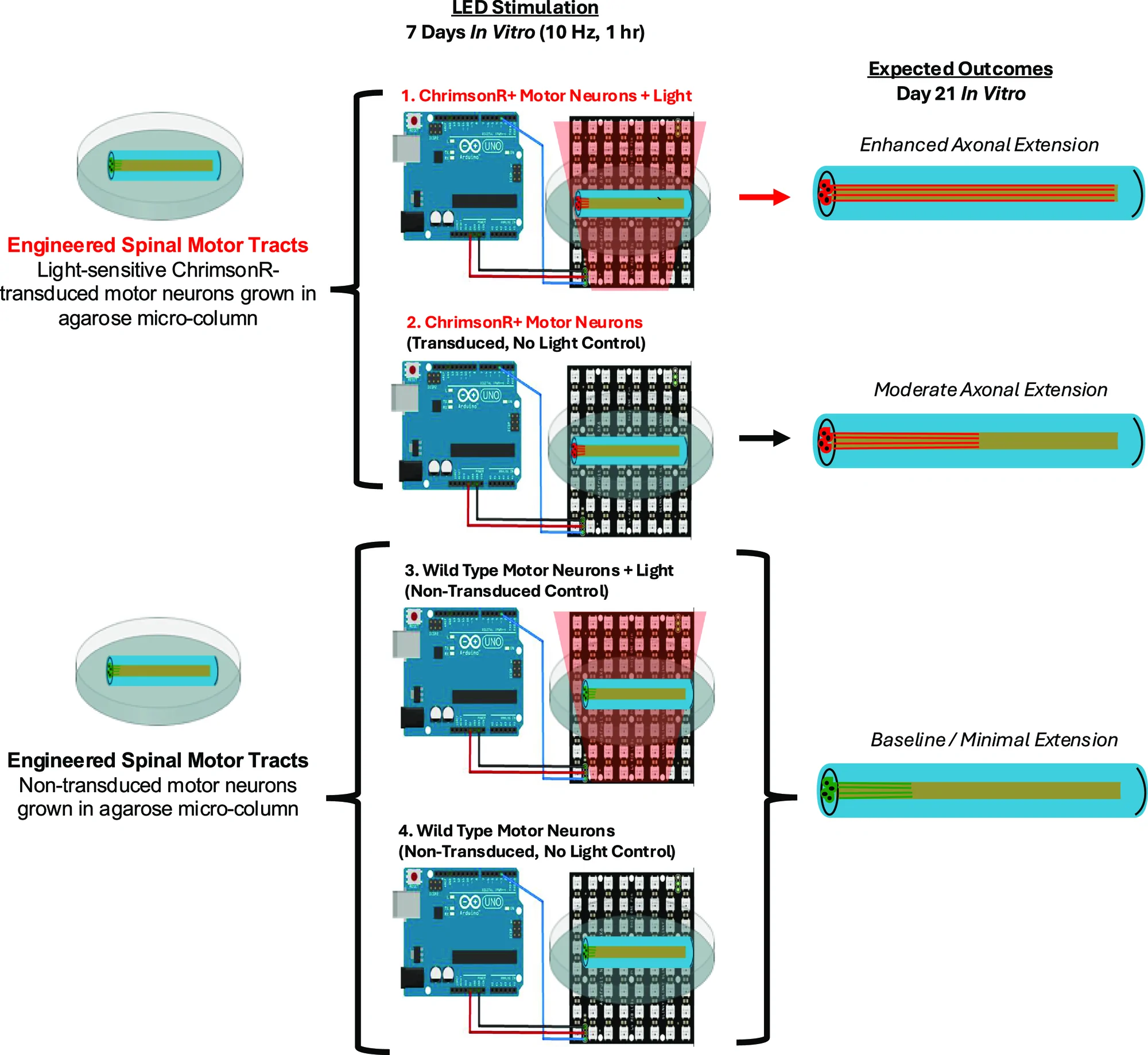
Optogenetic biomanufacturing paradigm for tissue-engineered motor axon tracts. Schematic illustrating the experimental framework to test whether optogenetic stimulation accelerates axonal extension in tissue-engineered spinal tracts (TE-STs), a subclass of micro–tissue-engineered neural networks (micro-TENNs) that recapitulate the architecture of white matter pathways. By integrating developmental neuroscience, biofabrication, and optogenetics, we evaluated four conditions: (1) *ChrimsonR^+^ motor neurons with light stimulation*: hypothesized to exhibit robust axonal extension; (2) *ChrimsonR^+^ motor neurons without stimulation*: expected to show some extension from ambient light; (3) *Non-transduced motor neurons with light stimulation*: not expected to show enhanced growth; and (4) *Non-transduced motor neurons without stimulation*—expected to show minimal extension. Together, these conditions test the hypothesis that targeted optogenetic activation is sufficient to drive accelerated axonal growth during *in vitro* biomanufacturing. This strategy is envisioned as particularly critical for producing otherwise difficult-to-grow tissues, such as motor neuron tracts, at clinically relevant scales, ultimately enabling off-the-shelf constructs for repairing devastating neurological injuries, including peripheral nerve and spinal cord damage.

## 2. Methods

All procedures were approved by the Institutional Animal Care and Use Committees at the University of Pennsylvania and adhered to guidelines set forth in the NIH Public Health Service Policy on Humane Care and Use of Laboratory Animals (2015).

### 2.1. TE-STs fabrication

Spinal MNs were isolated from embryonic day 16 Sprague-Dawley rats (Charles River) using an Optiprep density gradient methodology [25, 26, 35, 44]. MN aggregates were formed by adding 100 000 cells to a reverse pyramid well followed by centrifugation (1500 RPM for 5 min). Neurons were plated in spinal astrocyte-conditioned Neurobasal media +10% FBS supplemented with 37 ng ml^−1^ hydrocortisone, 2.2 μg ml^−1^ isobutylmethylxanthine, 10 ng ml^−1^ BDNF, 10 ng ml^−1^ CNTF, 10 ng ml^−1^ CT-1, 10 ng ml^−1^ GDNF, 2% B-27, 20 ng ml^−1^ NGF, 20 *μ*M mitotic inhibitors, 2 mM l-glutamine, 417 ng ml^−1^ forskolin, 1 mM sodium pyruvate, 0.1 mM *β*-mercaptoethanol, 2.5 g l^−1^ glucose. Neurons were transduced following centrifugation overnight with AAV2/1.hSynapsin.EGFP.WPRE.bGH or AAV.hSynapsin.tdTomato.

TE-STs were constructed using previously established micro-tissue engineering methodology to fabricate agarose micro-columns (3% agarose, 701 *µ*m outer diameter and 300 *µ*m inner diameter, 3–12 mm long) [25, 26, 32, 33, 39]. Micro-columns were UV-sterilized before adding 1 mg ml^−1^ rat tail Type 1 collagen + 0.1 mg ml^−1^ laminin, and crosslinking [39]. After polymerization, micro-columns were rinsed with 1x PBS and returned to the incubator overnight. MN aggregates were seeded on both ends of the micro-column with half media changes every other day. Cultures were maintained in phenol red–free media (90% Neurobasal medium + 10% fetal bovine serum).

### 2.2. Optical stimulation paradigm

Optical stimulation parameters were initially screened in planar spinal motor neuron cultures to enable rapid and controlled evaluation of stimulation conditions prior to application in three-dimensional constructs. Red light (625 nm) was delivered at 4 d *in vitro* (DIV) using an Arduino-controlled Pixel LED Matrix, with custom software providing independent control over illumination wavelength, spatial location, pulse duration, irradiance, and frequency. Power and pulse duration were first calibrated to ensure neuronal survival and normal morphology under repeated stimulation. Frequency was then evaluated using 1-second light pulses applied continuously for 1 h, with each well receiving a single assigned frequency. Optical spillover between wells was minimized by surrounding stimulated wells with unlit LEDs and enclosing the culture plate within a light-impermeable chamber.

This two-dimensional screening approach was selected for experimental efficiency and reproducibility, allowing multiple stimulation frequencies to be assessed under uniform conditions while minimizing confounds associated with three-dimensional tissue geometry. Based on trends observed in planar cultures, together with physiological relevance and tolerance considerations, a low-frequency stimulation paradigm was selected for subsequent three-dimensional studies.

TE-STs were then subjected to the selected stimulation parameters as a proof-of-concept assessment of biological effectiveness at 7 DIV for 1 h at 10 Hz and maintained in culture until fixation at 21 DIV. The 7 DIV stimulation point was chosen to allow neuronal aggregates to establish initial neurite outgrowth while remaining developmentally immature and responsive to activity-dependent cues. A single stimulation session was used to test whether a brief, programmable optical input could elicit sustained effects on axonal extension without imposing chronic stimulation burden during fabrication.

During three-dimensional stimulation, media temperature was monitored in a subset of cultures using a handheld infrared sensor positioned above the culture surface. No detectable temperature elevations were observed under the stimulation conditions used, confirming that the applied paradigm did not induce measurable heating artifacts. Together, these parameters were selected to be biologically effective and translationally appropriate for three-dimensional neural tissue biomanufacturing, while recognizing that further optimization directly within three-dimensional constructs represents an important direction for future studies.

### 2.3. Immunocytochemistry

Constructs were fixed with 4% paraformaldehyde (35 min), rinsed, and permeabilized (0.3% Triton X100 + 4% horse serum, 60 min) at various time points (1–21 DIV). Samples were incubated overnight at 4˚C with primary antibodies; axons were labeled with Tuj-1/beta-III tubulin (T8578, 1:500, Sigma-Aldrich) and MNs with choline acetyltransferase (ChAT, ab18376, 1:500, Abcam). Next, fluorescently tagged secondary antibodies (1:500; Invitrogen) were applied for 2 h at room temperature followed by a 10 min incubation with Hoechst (33342, 1:10000, ThermoFisher). Constructs were assessed at time points spanning 1–21 DIV.

### 2.4. Statistical analysis

Quantitative analyses were performed on neurite outgrowth metrics derived from both planar cultures and three-dimensional TE-STs. For planar cultures, neurite outgrowth was quantified as mean neurite area per aggregate. For TE-STs, axonal growth was quantified as mean neurite length along the construct axis.

To further assess longitudinal neurite propagation within TE-ST constructs, TUJ1+ signal was quantified at defined positions along the construct axis using region-of-interest (ROI)–based analysis of orthogonal z-stack reconstructions. Three ROIs were selected per construct corresponding to proximal (ROI A), mid (ROI B), and distal (ROI C) regions relative to the neuronal aggregate. For each ROI, TUJ1+ area was measured using consistent thresholding parameters across all samples. Longitudinal propagation of neurites was quantified at the sample level by calculating extension indices, defined as the ratio of TUJ1+ area in the mid or distal ROI to the proximal ROI. Specifically, mid-extension was calculated as ROI B divided by ROI A, and distal-extension was calculated as ROI C divided by ROI A. These indices normalize distal signal to proximal signal within the same construct, enabling comparison of neurite propagation independent of absolute signal intensity. ROI-level measurements were treated as technical subsamples and aggregated at the construct level prior to statistical analysis.

In all experiments, a single neuronal aggregate was placed in each well or TE-ST construct, and each aggregate or construct was treated as an independent biological replicate. For each experimental condition, mean values were calculated from *N* = 3–4 independent aggregates or constructs, as indicated in the corresponding figure legends. Data are presented as mean ± standard error of the mean (SEM). Statistical comparisons were performed using two-way ANOVA with factors for stimulation and transduction, followed by Tukey’s multiple comparisons test where appropriate. Statistical significance was defined as p < 0.05.

## 3. Results

### 3.1. TE-ST characterization *in vitro*

TE-STs spanning 3–12 mm comprised of two MN aggregates on either end of an agarose micro-column (701 *µ*m outer diameter and 300 *µ*m inner diameter) were fabricated using variations of previously established micro-tissue engineering techniques [25, 26, 29–33, 39]. Healthy motor neuron aggregates lacking a necrotic core with dense axonal projections extending through the lumen were readily visualized through the transparent micro-column (figure 2(A)). TE-STs could be readily fabricated at varying lengths (figures 2(A) and (B)). Discrete populations of ChAT+ MNs with Tuj1+ axons extending the length of the micro-column were visualized within the micro-column (figure 2(C)). Notably, increasing the inter-aggregate distance (via longer micro-columns) slowed the rate of axonal extension. For example, 10-mm TE-STs containing one or two neuronal aggregates at opposite ends (∼8–9 mm inter-aggregate distance) rarely exceeded 2 mm of axonal growth by 21 DIV. In contrast, shorter TE-STs exhibited greater axon density and tighter fasciculation at comparable time points. Despite these differences in axonal growth, neuronal health appeared unaffected by micro-column length. These findings suggest that inter-aggregate distance influences the growth and maturation of axonal micro-tissue *in vitro*.

**Figure 2.**
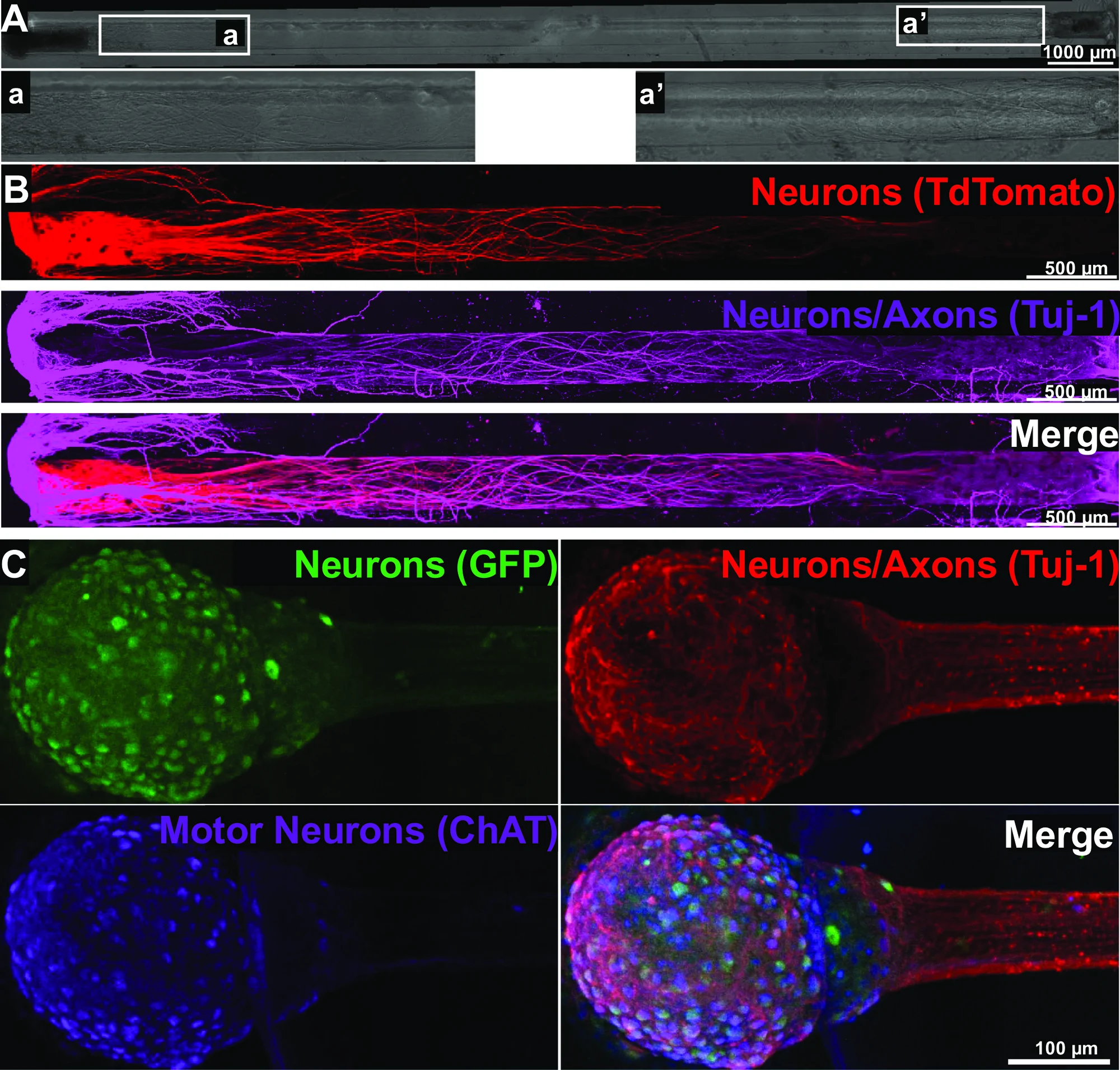
Fabrication and characterization of tissue-engineered spinal tracts (TE-STs). TE-STs are anatomically inspired, three-dimensional micro-tissues composed of discrete motor neuron populations extending aligned axonal tracts within the lumen of an agarose micro-column. The constructs can be fabricated at variable lengths depending on the intended application. (A) Representative phase-contrast images of a 1 cm bi-directional TE-ST at 14 d *in vitro* (DIV), showing neuronal populations at each end of the micro-column (insets a, a′). (B) Confocal images of a 5 mm bi-directional TE-ST at 7 DIV, with one neuronal population transduced to express TdTomato (red) and co-labeled for β-III-tubulin (Tuj1, magenta). Axons extend robustly through the lumen, crossing the midline by 7 DIV, whereas few if any cross the 1 cm distance by 14 DIV. (C) Immunocytochemistry of TE-STs at 7 DIV demonstrating discrete populations of ChAT-positive (ChAT^+^) motor neurons (purple) and Tuj1^+^ axons (red). Neurons were transduced to express GFP (green) for visualization. High-resolution confocal imaging confirms co-localization of Tuj1^+^/ChAT^+^/GFP^+^ motor neurons and aligned axonal projections along the micro-column. Nuclei were counterstained with Hoechst (blue). Scale bars: A, 1000 *µ*m; B, 500 *µ*m; C, 100 *µ*m.

## 4. Optogenetic stimulation

Optogenetics enables cell-type–specific, light-controlled depolarization through the expression of channelrhodopsins, providing a precise means of regulating neuronal activity [45]. In contrast to electrical stimulation, which non-specifically activates mixed populations, optogenetic stimulation allows selective and transient activation of targeted neurons. Previous work has shown that this approach can enhance growth factor secretion and promote axonal outgrowth in sensory neurons [40]. To adapt this strategy for spinal MNs, stimulation parameters were initially optimized in planar culture. After calibrating light power and duration, motor neuron aggregates expressing channelrhodopsin were exposed to 1 h of red-light stimulation at 4 DIV across a range of frequencies (0–130 Hz), and neurite outgrowth was quantified at 7 DIV (figure 3).

**Figure 3.**
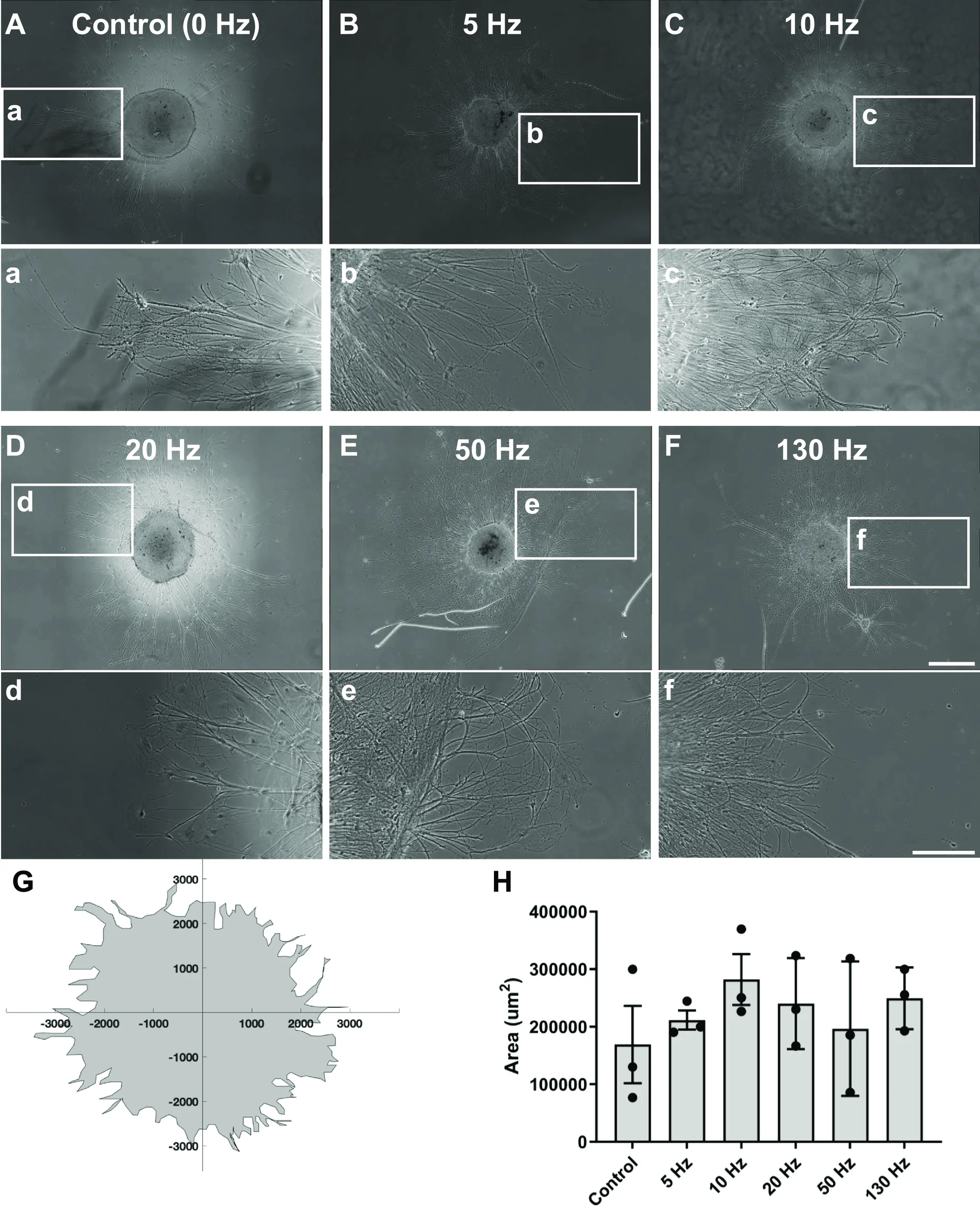
Optimization of Optogenetic Stimulation Frequency for Motor Neuron Outgrowth. (A)–(F) Representative phase-contrast images of embryonic rodent motor neuron aggregates transduced with channelrhodopsin, imaged at 7 DIV following a 1 h light stimulation on 4 DIV at the indicated frequencies. (a)–(f) Magnified regions from white boxes highlight differences in neurite outgrowth. (G) Quantification of neurite field area using a custom MATLAB script. (H) Summary of neurite outgrowth across stimulation frequencies, presented as mean ± SEM (*n* = 3 aggregates per condition). Among the frequencies tested, 10 Hz produced the largest neurite outgrowth area in this planar culture paradigm. Scale bars: 500 *µ*m (A)–(F); 250 *µ*m (a)–(f).

Although no individual stimulation frequency reached statistical significance relative to others in the planar screening assay, 10 Hz was selected based on a convergence of empirical trend, physiological relevance, and translational practicality. In two-dimensional cultures, 10 Hz consistently produced the largest neurite field area with low inter-sample variability. Importantly, this frequency lies within the physiological firing range reported for spinal MNs during sustained motor output and tonic activation, making it a biologically relevant stimulation regime. Higher frequencies did not confer a clear growth advantage in planar screening and were therefore not prioritized for subsequent three-dimensional testing (Data Not Shown).

To assess whether optogenetic stimulation could accelerate axonal growth in TE-STs, motor neuron aggregates were cultured within agarose micro-columns and transduced to express ChrimsonR. At 7 DIV, constructs were exposed to a single 1 h round of 10 Hz light stimulation using a custom LED system. At 8 DIV (one day after stimulation), axonal lengths did not differ significantly between groups (Transduced + Stimulation: 2029.4 ± 95.7 *µ*m; Transduced + No Stimulation: 2007.4 ± 67.7 *µ*m). However, by 11 DIV, transduced constructs that had received stimulation exhibited markedly greater axonal extension compared to non-stimulated transduced controls (3422.2 ± 243.1 *µ*m vs. 2316.8 ± 186.6 *µ*m; *p* < 0.05), an effect that persisted throughout subsequent days in culture (figure 4). This delayed divergence suggests that stimulation triggers secondary processes, such as transcriptional or signaling cascades, that sustain axon elongation beyond the acute stimulation period, which could be leveraged to shorten biomanufacturing timelines.

**Figure 4.**
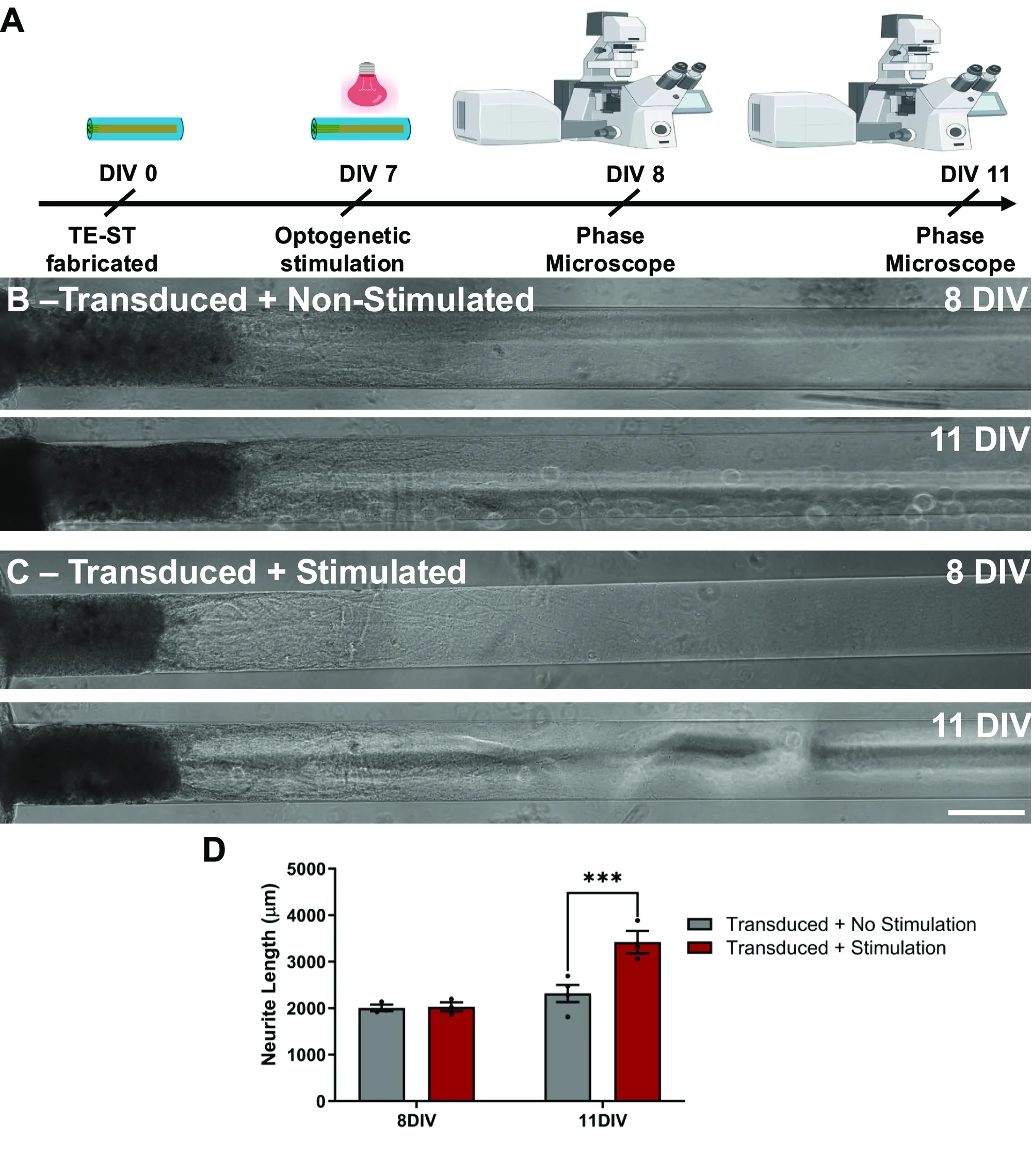
Engineered Spinal Motor Neurite Growth Following Light Stimulation. (A) Tissue-engineered spinal tracts (TE-STs) composed of spinal motor neurons transduced to express the red-shifted channelrhodopsin ChrimsonR were stimulated with red light (10 Hz, 1 h) at 7 d *in vitro* (DIV). Neurite outgrowth was assessed by phase-contrast microscopy at 8 and 11 DIV. (B), (C) Representative phase-contrast images over time show motor neurites extending from discrete neuronal populations within hydrogel micro-columns, resembling organized *in vivo*-like architecture. (D) Quantification of neurite length in TE-STs at 8 and 11 DIV. At 8 DIV (1 d after stimulation), no significant differences in neurite length were observed between stimulated and non-stimulated constructs. By 11 DIV, stimulated TE-STs exhibited a marked increase in neurite outgrowth relative to controls. Data are presented as mean ± SEM (n = 3 TE-STs per condition). Statistical comparisons were performed using two-way ANOVA, with stimulation and transduction treated as independent variables (*p* < 0.05; *p < 0.01; **p < 0.001). Scale bar: 500 *µ*m.

At 21 d *in vitro*, confocal reconstructions supported the quantitative analyses, revealing long, fasciculated axon bundles spanning the constructs, with Tuj1^+^ axons extending from ChrimsonR^+^ MNs (figure 5). Quantification showed that the non-transduced, non-stimulated baseline group exhibited the shortest outgrowth (2010.0 ± 420.9 *µ*m; 1.0× baseline). Non-transduced neurons exposed to light stimulation showed no improvement (2873.2 ± 167.2 *µ*m; 1.4× baseline, ns vs. baseline). Transduced neurons without stimulation extended significantly further than both non-transduced groups (3976.7 ± 215.4 *µ*m; 2.0× baseline; *p*< 0.05). The greatest effect was observed in transduced neurons exposed to light stimulation, which reached 5319.3 ± 135.8 *µ*m (∼2.3× baseline; *p* < 0.0001 vs all other groups). Two-way ANOVA confirmed main effects of both transduction and stimulation, with a strong interaction between the two variables. ChrimsonR expression alone produced a modest increase in axonal outgrowth, consistent with a baseline effect of transduction and potentially incidental light exposure from routine imaging.

**Figure 5.**
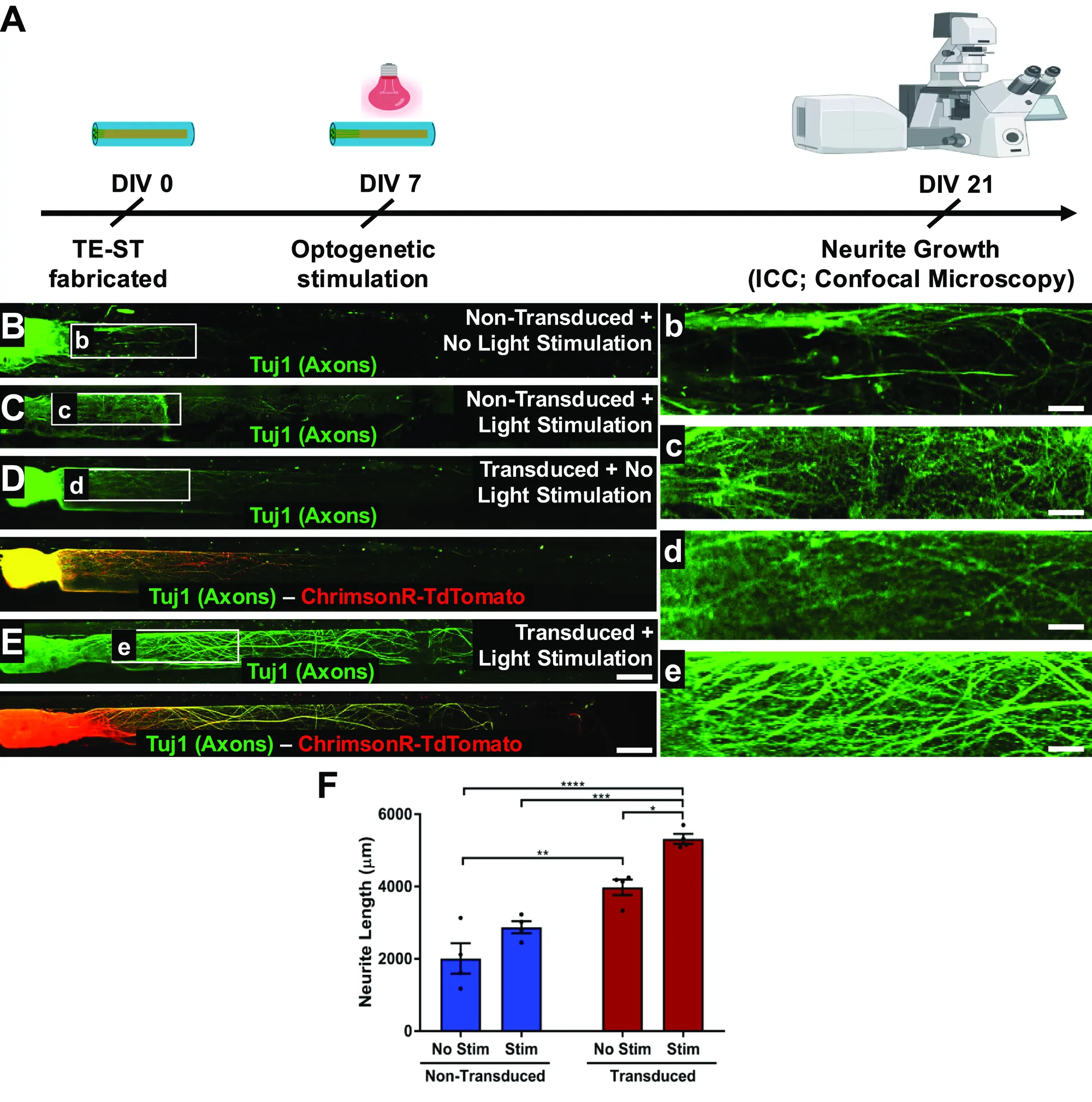
Optogenetic stimulation enhances axonal outgrowth in three-dimensional motor neuron constructs. (A) Schematic of the experimental design. Spinal motor neurons were transduced to express ChrimsonR, a red-shifted channelrhodopsin tagged with TdTomato (red) for visualization. At 7 d *in vitro* (DIV), constructs were stimulated with red light (10 Hz, 1 h). Control groups were maintained under identical conditions without stimulation. (B)–(E) Representative confocal reconstructions of tissue-engineered spinal tracts (TE-STs) at 21 DIV. Axons were labeled with β-III-tubulin (Tuj1, green), and ChrimsonR–TdTomato expression is shown in red. Insets (b)–(e) show higher-magnification views of axonal tracts within the column lumen. (F) Quantification of neurite length demonstrated significantly greater outgrowth in transduced TE-STs receiving light stimulation compared with transduced non-stimulated controls and with both non-transduced groups. Data are presented as mean ± SEM (*n* = 3 TE-STs per condition). Statistical comparisons were performed using two-way ANOVA, with stimulation and transduction treated as independent variables (*p* < 0.05; *p < 0.01; **p < 0.001). Scale bars: 500 *µ*m (B–E); 25 *µ*m (b)–(e).

Additional assessment of neurite propagation within three-dimensional TE-ST constructs was performed by quantifying TUJ1+ signal at defined positions along the construct axis using ROI-based analysis of orthogonal reconstructions (figure 6). While proximal neurite presence was observed across all conditions, marked differences emerged in distal longitudinal extension. Stimulated, transduced constructs exhibited significantly greater neurite propagation to both mid and distal regions compared to all other groups. Mid-extension (ROI B/ROI A) was increased in stimulated transduced constructs (0.9 ± 0.3) relative to stimulated non-transduced (0.2 ± 0.2) and non-stimulated controls (0.05 ± 0.01), with this effect further amplified at the distal region, where distal-extension (ROI C/ROI A) was significantly higher than all other conditions (0.5 ± 0.1 vs 0.1 ± 0.03 and 0.06 ± 0.02 for stimulated and non-stimulated non-transduced controls, respectively).

**Figure 6.**
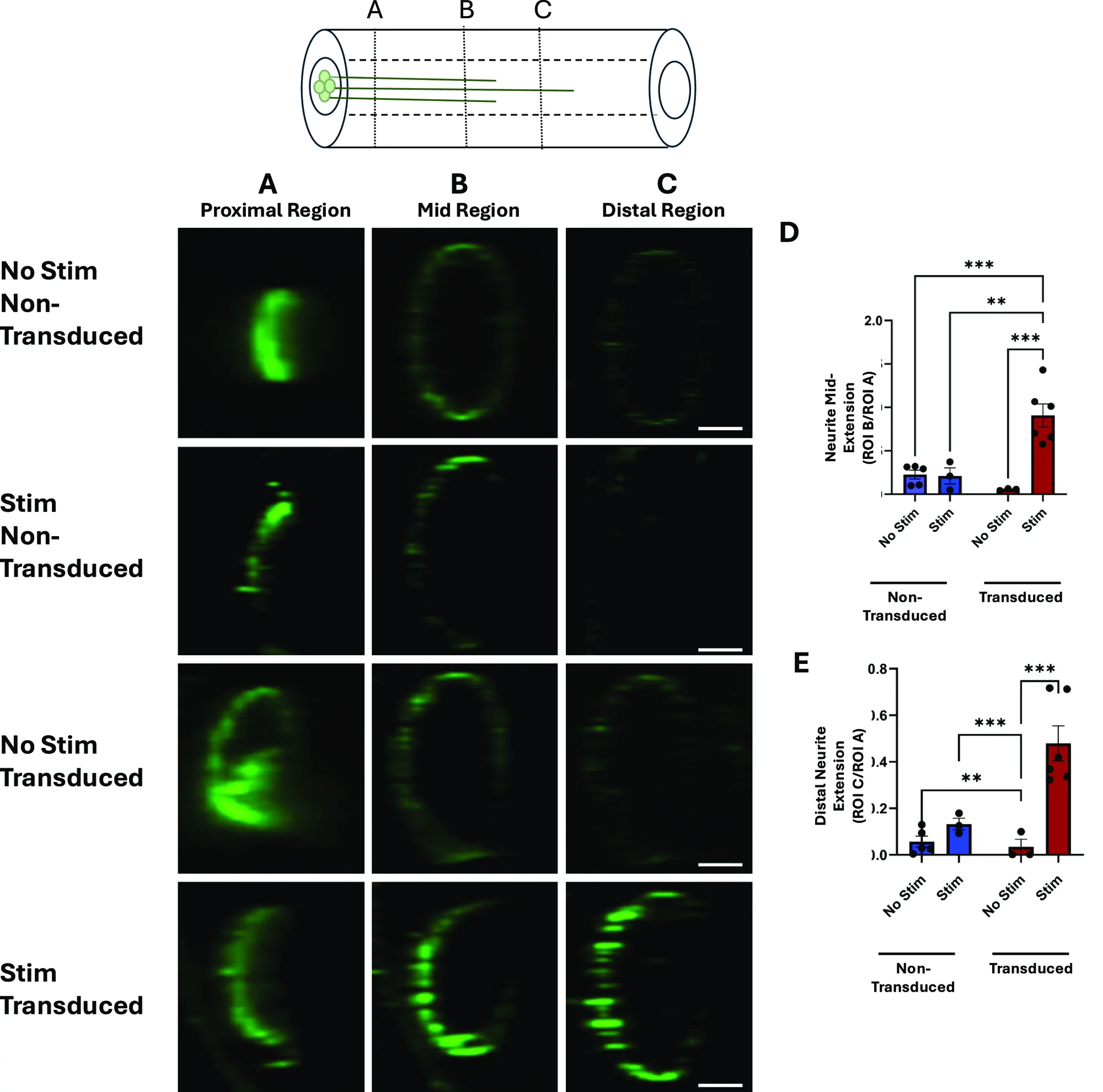
Longitudinal extension of TUJ1+ neurites along engineered constructs.(A)–(C) Representative orthogonal reconstructions showing TUJ1+ signal distribution across proximal (A), mid (B), and distal (C) regions of the construct under indicated stimulation and transduction conditions. Quantification of neurite extension was performed using region-of-interest (ROI)–based analysis along the construct axis. Mid-extension (ROI B/ROI A) and distal-extension (ROI C/ROI A) indices were calculated for each sample. DATA are shown as individual samples with mean ± SEM. Two-way ANOVA revealed significant effects of stimulation and transduction, with a strong interaction between factors. Stimulated transduced constructs exhibited significantly greater mid- and distal-extension compared to all other groups. A cartoon schematic illustrating the location of the ROIs is shown at the top. Scale bars, 75 *µ*m*. p* < 0.05; *p < 0.01; **p < 0.001; ***p < 0.0001.

In contrast, non-transduced constructs demonstrated limited propagation beyond the proximal region, with low mid- and distal-extension indices. Notably, ChrimsonR-transduced MNs exhibited a modest but reproducible increase in axonal extension relative to non-transduced controls even in the absence of programmed optical stimulation (0.03 ± 0.03 vs 0.06 ± 0.02), consistent with low-level opsin activation during routine handling or baseline effects of viral transduction. However, this baseline enhancement was substantially smaller than that observed with patterned stimulation and did not account for the magnitude of the growth phenotype.

Exposure of non-transduced neurons to identical red-light stimulation did not enhance axonal extension, indicating that light alone was insufficient to drive growth. Consistent with this, illumination was delivered at low irradiance using a red-shifted wavelength, and direct temperature measurements showed only minimal increases (⩽0.7 °C), remaining within physiological limits (supplementary table 1). Together, these findings support an activity-dependent mechanism requiring both opsin expression and optical stimulation, rather than nonspecific thermal or photochemical effects.

## 5. Discussion

The present study demonstrates that optogenetic stimulation can be harnessed as a biofabrication tool to accelerate the assembly of tissue-engineered spinal motor tracts (TE-STs). Applying patterned optical stimulation within hydrogel micro-columns produced an approximately 2.5-fold increase in axonal outgrowth, enabling long spinal motor tracts to form within shorter culture periods than previously achievable. Accordingly, the stimulation parameters applied here should be viewed as biologically effective and translationally appropriate rather than necessarily optimal for three-dimensional constructs. Future studies will be required to systematically refine stimulation regimes directly within three-dimensional tissues.

Consistent with prior work showing that optogenetics enables precise tuning of stimulation parameters such as frequency, pulse duration, and intensity, the observed enhancement was frequency-dependent. These findings align with reports from three-dimensional motor neuron–Schwann cell cocultures, where 20 Hz stimulation modestly enhanced neurite outgrowth and myelination over several weeks. Together, these results reinforce the principle that activity-driven mechanisms can be exploited to accelerate axonal elongation and maturation during tissue fabrication. This study builds on earlier work using preformed neuronal networks for neural repair [28, 30, 33] by introducing an optogenetic approach that directly enhances intrinsic neuronal growth programs.

Optogenetic stimulation has also been shown to increase axonal growth in other neuronal populations. For example, activation of DRG in two-dimensional culture increased neurite extension and secretion of trophic factors such as BDNF and GDNF [40]. Moreover, wild-type DRG axons extended more rapidly when co-cultured with optogenetically active DRG, a phenomenon described as AFAR [35, 36]. The present results extend these observations to spinal MNs in a unidirectional, three-dimensional context, underscoring that activity-dependent mechanisms can be leveraged to generate long, aligned axon tracts suitable for transplantation.

Previously, engineered micro-tissues containing spinal MNs at one or both ends of hydrogel micro-columns functioned as neuromuscular interfaces, driving muscle contraction for up to 16 weeks after peripheral nerve injury in the absence of host reinnervation [25]. Motor axons were also shown to extend well beyond the typical 2 mm limit *in vitro* when co-cultured with aligned Schwann cells [26]. The present findings expand on this work by demonstrating that optogenetic stimulation accelerates axonal elongation during biomanufacturing.

The enhancement of axonal growth observed here is consistent with prior studies showing that neuronal activity accelerates outgrowth in permissive environments. In both sensory and cortical neurons, opto- and chemogenetic activation increased neurite extension *in vitro* and promoted sciatic nerve regeneration *in vivo*, whereas this effect was lost after SCI due to inhibitory chondroitin-sulfate proteoglycans [46]. These results highlight that activity-dependent growth requires a supportive extracellular context, recapitulated here by the collagen matrix confined within agarose micro-columns. Although the ChrimsonR opsin used in this work primarily mediates general cation conductance rather than direct calcium influx [47], newly developed channelrhodopsins with enhanced Ca^2+^ permeability demonstrate that optical activation can directly engage intracellular calcium signaling [48]. Together, these findings suggest that optogenetic stimulation promotes axonal elongation through complementary mechanisms involving membrane depolarization, calcium signaling, and transcriptional activation when inhibitory cues are minimized. While this study was not designed to directly interrogate downstream molecular signaling, several observations support an activity-dependent growth mechanism. The absence of immediate differences following stimulation, followed by sustained divergence days later, is consistent with delayed transcriptional or signaling cascades downstream of neuronal depolarization rather than acute cytoskeletal effects. Additionally, the requirement for both opsin expression and patterned optical stimulation, together with the absence of effects in non-transduced controls, argues against nonspecific photothermal or viral artifacts. Future studies incorporating calcium imaging, immediate-early gene expression, or phospho-signaling analyses will be necessary to directly validate the molecular pathways engaged by optogenetic activation in this biomanufacturing context.

This work extends earlier demonstrations that optogenetic activation enhances neurite elongation in two-dimensional sensory cultures to a three-dimensional engineered tissue format. The observed acceleration of motor axon growth is consistent with previous evidence that calcium-dependent signaling supports cytoskeletal remodeling and gene activation during elongation [46, 48]. Incorporating programmable optical stimulation into the fabrication workflow establishes optogenetic activation as a quantitative, precisely controllable input [49], linking neuronal activity directly to tissue development. Within this platform, neurons extended through a permissive collagen matrix enclosed in agarose micro-columns, and further optimization of scaffold composition—such as aligned or degradable architectures, growth-factor gradients, or conductive polymers—may refine optical penetration, excitability, and mechanical compliance during fabrication. Our data also complement scaffold-based strategies for neural repair, including aligned nanofiber or hydrogel channels that promote directional regrowth across lesion sites [50], and parallel the biological relay formation achieved through neural progenitor grafts that restore corticospinal continuity in preclinical models [51]. Future iterations could incorporate conductive or adaptive hydrogel matrices to facilitate long-term electrical coupling and tissue integration, aligning with advances in implantable hydrogel interfaces for next-generation brain–computer technologies [52].

Compared with self-organized organoid systems [53, 54], the optogenetically fabricated tracts described here provide externally tunable activity patterns that can be standardized across batches, addressing reproducibility challenges in complex three-dimensional models [55]. Optical stimulation therefore serves not only as a tool for neural activation but also as a programmable parameter within the biomanufacturing process. Advances in patterned illumination and multimodal optical control now enable spatiotemporal regulation of neuronal activity with subcellular precision [49], allowing defined activity profiles to be integrated directly into fabrication workflows. Light-based biofabrication approaches have shown that controlled illumination can guide tissue organization and maturation across diverse cell types [56], suggesting that similar strategies may accelerate and standardize the assembly of engineered neural tracts.

Integrating optical or electronic stimulation interfaces during or after implantation could further modulate graft activity and promote functional recovery, as demonstrated by recent wireless nanoelectronic systems developed for neural repair [57]. Emerging industrial efforts are likewise embedding optogenetic stimulation directly into biohybrid devices to enable real-time control of living neural interfaces [58, 59]. Together, these advances highlight a convergent trajectory toward scalable, optically regulated biomanufacturing of functional neural tissue constructs for translational repair and neuroprosthetic applications.

Although the present study was confined to *in vitro* analyses, these findings highlight several important translational opportunities. Optogenetic stimulation has been shown to promote motor axon regeneration across inhibitory spinal cord lesions in zebrafish and to increase axonal sprouting proximal to injury sites in mammalian models [46, 60]. However, in the absence of structural support, these activity-driven axons typically fail to traverse the proteoglycan-rich lesion core. Engineered neural tissues offer distinct advantages over dissociated cell injections or acellular scaffolds by providing defined cytoarchitecture that enhances cell survival, enables predictable connectivity, and aligns structural design with mechanism of action through guided axonal growth. [28, 30, 33] Following SCI, the formation of a large fluid-filled cavity precludes sustained cell survival and host axon regeneration; TE-STs may overcome this limitation by providing a preformed axonal pathway that bridges the lesion cavity and directly connects rostral and caudal spinal parenchyma, creating a living substrate for host axon guidance. With further optimization, such constructs may also promote synaptic integration and circuit-level plasticity. Importantly, the manufacturing paradigm permits pre-implantation assessment of construct structure, viability, and potency, which is essential for translational readiness and regulatory compliance.

The present work utilized embryonic rat MNs due to their robustness, reproducibility, and established performance within the TE-ST platform; however, differences between embryonic rodent neurons and human iPSC-derived MNs warrant consideration for clinical translation. Human neurons exhibit distinct maturation timelines, excitability thresholds, and viral transduction efficiencies that may influence responsiveness to optical stimulation. Nevertheless, the optogenetic biomanufacturing framework described here is cell-source agnostic, and prior work has demonstrated the feasibility of generating long-projecting axon tracts from human iPSC-derived neurons. Future studies will directly evaluate whether optical stimulation similarly accelerates axonal growth and maturation in human cell–based constructs, representing a critical step toward translational deployment.

A limitation of the present study is that stimulation parameters were optimized in two-dimensional cultures before being applied to three-dimensional TE-ST constructs. Stimulation parameters were initially screened in two-dimensional cultures for experimental convenience and efficiency, enabling rapid evaluation across a broad frequency range under controlled conditions. These parameters were then subjected to cursory validation in three-dimensional TE-ST constructs to assess basic construct tolerance and neuronal health rather than formal optimization. Using this approach, lower-frequency stimulation was consistently well tolerated in three-dimensional constructs and supported robust axonal extension. Notably, preliminary pilot testing indicated that high-frequency stimulation paradigms (e.g. 130 Hz) were poorly tolerated in three-dimensional TE-STs, with reduced neuronal health and disrupted aggregate morphology relative to lower-frequency stimulation. Although the underlying mechanism was not systematically investigated, this observation suggests that increased effective stimulation load (frequency × duration) may negatively impact neuronal viability within collagen-based three-dimensional microenvironments. These findings further motivated the selection of a conservative stimulation frequency for the present study. Although the agarose micro-columns and collagen matrix are optically permissive and confine axons within the narrow lumen, neurons within micro-columns may respond differently to optogenetic drive due to differences in tissue geometry, photon distribution, nutrient diffusion, or network connectivity. In addition, while the observed delayed and sustained enhancement of axonal growth is consistent with activity-dependent signaling, this study did not directly interrogate downstream molecular mechanisms, such as calcium dynamics or transcriptional activation. Another limitation is the use of embryonic rat MNs, which may not fully recapitulate the responses of human iPSC-derived neurons intended for clinical translation, particularly with respect to maturation kinetics, viral transduction efficiency, and culture sensitivity. Future studies should refine stimulation paradigms directly in three-dimensional constructs, incorporate spatially distributed or multi-site illumination, evaluate human cell sources, and integrate molecular or functional readouts to further enhance axonal extension, maturation, and translational readiness. Moreover, combining optogenetic stimulation with engineered tissue may enable advanced biohybrid interfaces for bidirectional, synaptic-level modulation [58].

This study demonstrates optogenetic stimulation may activate regenerative mechanisms that drive long axon extension *in vitro*. The constructs described here, which recapitulate key features of descending spinal pathways, provide a strategy for generating mature neural tissues with preformed axons suitable for translational deployment. Future work should evaluate optogenetic stimulation during the biomanufacturing of centimeter-scale constructs and determine whether post-transplant stimulation can accelerate graft integration while enabling external modulation of graft activity and downstream targets in vivo. Collectively, these findings position optogenetically stimulated TE-STs as a promising platform for scalable biofabrication and restoration of long-distance connectivity after neurotrauma. Future studies incorporating automated fabrication, stimulation, and quantitative detection pipelines will be essential to validate these findings at scale, assess batch-to-batch variability, and define robust operating windows for optogenetic biomanufacturing of engineered neural tissues.

## Acknowledgments

The authors thank Lilly Ryll, Rose Horowitz, and Darryl Previlor for technical contributions. Financial support was provided by the National Institutes of Health [R01-AR083489 (Cullen), R01-NS127895 (Cullen), TL1-TR001880 (Burrell), T90-DE030854 (Burrell)], the Department of Veterans Affairs [I01-RX005045 (Cullen)], and Penn’s University Research Foundation (Ozturk).

## Data Availability

The data cannot be made publicly available upon publication because no suitable repository exists for hosting data in this field of study. The data that support the findings of this study are available upon reasonable request from the authors.

## Appendix

**Supplemental Table 1.** Effect of light stimulation on media temperature. Light activation has been reported to impact nerve regeneration potentially via temperature-sensitive protein channels. In this study, a simulated stimulation paradigm was used to measure temperature over time. Media dishes were illuminated from a custom light-emitting diode for 1 h with a frequency of 10 Hz. Temperature was sampled every 15 min starting at baseline and ending at 60 min. Although illumination produced a modest rise over 60 min (⩽0.7 °C vs. control), the peak value (∼37 °C) was within physiological temperature; two-way ANOVA detected no effect of light or time.

Conditions.	Baseline.	15 min.	30 min.	60 min.
Illuminated (*n*=3)	35.4 °C ± 0.1 °C	36.4 °C ± 0.3 °C	36.8 °C ± 0.3 °C	37.0 °C ± 0.5 °C;
Control (*n*=3)	35.3 °C ± 0.2 °C	36.0 °C ± 0 °C	36.1 °C ± 0.1 °C	36.3 °C ± 0.1 °C
